# Cell Therapy Augments Functional Recovery Subsequent to Spinal Cord Injury under Experimental Conditions

**DOI:** 10.1155/2015/132172

**Published:** 2015-07-09

**Authors:** Vikram Sabapathy, George Tharion, Sanjay Kumar

**Affiliations:** ^1^Centre for Stem Cell Research, Christian Medical College, Bagayam, Vellore, Tamil Nadu 632002, India; ^2^Department of Physical Medicine and Rehabilitation, Christian Medical College, Vellore, Tamil Nadu 632002, India

## Abstract

The spinal cord injury leads to enervation of normal tissue homeostasis ultimately leading to paralysis. Until now there is no proper cure for the treatment of spinal cord injury. Recently, cell therapy in animal spinal cord injury models has shown some progress of recovery. At present, clinical trials are under progress to evaluate the efficacy of cell transplantation for the treatment of spinal cord injury. Different types of cells such as pluripotent stem cells derived neural cells, mesenchymal stromal cells, neural stem cells, glial cells are being tested in various spinal cord injury models. In this review we highlight both the advances and lacuna in the field of spinal cord injury by discussing epidemiology, pathophysiology, molecular mechanism, and various cell therapy strategies employed in preclinical and clinical injury models and finally we discuss the limitations and ethical issues involved in cell therapy approach for treating spinal cord injury.

## 1. Introduction

Spinal cord injury (SCI) is a serious debilitating disorder that results in complete or partial loss of motor/sensory neuronal functions due to mechanical damage of the spinal cord [1]. Overall analysis of the incidence report suggests that extent of patients suffering from spinal cord injury might approximately vary from 8 to 83 cases per million factoring into account diversities in geographical and socioeconomic and political conditions [2–4]. The spinal cord injury can be broadly classified into two groups: traumatic and nontraumatic [3]. Traumatic spinal cord injury results from contusion, compression, and stretch of the spinal cord [5]. Trauma related injury is the most prevalent among SCI cases majorly involving road traffic accidents, especially in case of young adults between age group of 15 and 29 years and accidental falls in case of aged people (>65 years) [6, 7]. Nontraumatic related injury mainly consists of vertebral spondylosis, tumor compression, vascular ischemia, and congenital and inflammatory spinal cord disorders [8]. Several different treatment strategies such as drug intervention (steroidal/nonsteroidal), growth factors, cellular metabolites (cAMP/GTPases), small molecules, extracellular matrices, and cellular therapy involving pluripotent stem cells/mesenchymal stem cells (MSCs)/neural progenitor cells (NPCs/NSCs) are being tested for successful therapeutic intervention [9]. Incidentally, various therapeutic strategies exist to alleviate the symptoms/complications but there is no proper treatment available to completely cure spinal cord injury.

## 2. Physiological**  **Complications due to Spinal Cord Injury

**Figure 1:**
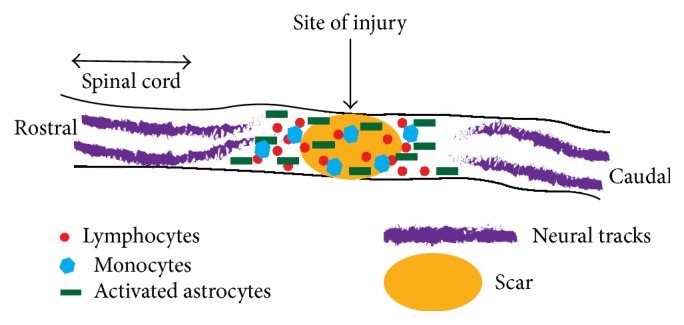
Mechanism of spinal cord injury.

The pathophysiological stages after spinal cord injury can be classified into primary and secondary phases [10, 11]. The primary phase is the phase at the moment of aberration in spinal cord structure due to mechanical forces. The spinal cord at the time of injury may be subjected to hyperbending, overstretching, twisting, or laceration [12]. The complications arising in the secondary phase are directly proportional to the extent of injury in the primary phase. The secondary phase can be in turn classified into three different subphases such as acute phase (2 hours to 2 days), subacute phase (days to weeks), and chronic phase (months to years) [13–15]. The inflammatory response mediated by convoluted cellular and molecular interactions after spinal cord trauma forms the core of secondary injury phase. The acute phase is characterized by edema, ischemia, hemorrhage, reactive oxygen species (ROS) production, lipid peroxidation, glutamate mediated excitotoxicity, ionic dysregulation, blood-spinal cord barrier permeability, inflammation, demyelination, neuronal cell death, and neurogenic shock. The subacute phase is comprised of activation and recruitment of microglial cells, astrocytes, monocytes, T lymphocytes, and neutrophils, macrophage infiltration, scar formation, and initiation of neovascularization. The chronic phase exhibits neuronal apoptosis, retraction and demyelination of axons, loss of sensorimotor functions, Wallerian degeneration, glial scar maturation, cyst and syrinx formation, cavity formation, and Schwannosis [16, 17] (Figure 1). The subacute phase after spinal injury provides optimal time frame for therapeutic interventions [18].

## 3. Molecular Mechanism of Spinal Cord Injury

The trauma of spinal cord injury results in an irreversible and progressive degeneration of neuronal tissue. After spinal cord injury, the acute and chronic phases are accompanied by various molecular changes leading to inflammation, loss in biochemical homeostasis, and degeneration of neurofilaments, higher ROS (reactive oxygen species) levels and apoptosis [1]. During the onset of spinal cord injury various injury genes are activated. Based on the meta-analysis of the previous reports, these genes can be broadly classified into early and late injury genes depending upon the phase of activation or downregulation [1]. The first 24–48 hours refers to early injury phase and late phase represents 1 week after injury. Molecular cascade after spinal cord injury results in the activation of genes responsible for inflammatory pathway, apoptosis, cell cycle and oxidative stress, and downregulation of genes involved in energy metabolism, lipid metabolism, neurotransmission, and cytoskeleton [1]. Inflammation is a convoluted process. It can be broadly classified into acute and chronic inflammatory. Immediately after spinal cord injury, the proinflammatory cytokines such as IL6, TNF*α*, and IL1*β* are activated and expressed multifold [90, 91]. Apoptosis refers to programmed cell death. As a result of spinal cord injury, it has been observed that there is significant increase in the proapoptotic genes such as Bad, Bax, p53, AFAP1, caspase 3, and caspase 9, during the early phase of injury and significant decrease in antiapoptotic genes like Bag1 and Bcl2 during late phase of injury [90, 92, 93]. Following spinal cord injury there is elevation of cell cycle associated genes such as PCNA, cyclin D1, cdk4, cyclin G, Rb, E2F5, c-Myc, and Gadd45a which might support the neuronal cell death [94]. Further, reports suggest that spinal cord injury results in the redox imbalance in the tissues leading to increased reactive oxygen species (ROS) levels and oxidative stress. Consequently, upregulation of Hspb1 [90] and Hspa4 [95] genes involved in regulating oxidative stress was observed during the early phase of injury. Lipid metabolism in general involves lipogenesis and fatty acid oxidation. It is required to maintain the tissue homeostasis. During the later phase of injury it is observed that genes involved in lipid biosynthesis such as Gpd1 and Alox12 required for lipooxygenase are effectively downregulated [92]. Most importantly, genes involved in neural excitation such as Kcnh2/k1/c1, Scn1a/8a, Gria3, and Grm3, neurotransmission related genes like Gabra5/b1, synapse associated genes like Slc6a1, and genes required to maintain electrochemical gradient such as Atp1a3 and Atp2a1/b2 are found to be markedly downregulated during both early and late phases of spinal cord injury [95, 96]. Also cytoskeleton-scaffolding proteins like Nef1 required for maintenance of axonal cytoskeleton and Map2 involved in microtubule assembly are notably downregulated [95].

## 4. Cell Therapy

Currently, there are limited therapeutic interventions and no proper cure for effective treatment of spinal cord injury but recent preliminary studies have indicated that cell therapy may help. For effective functional recovery, the transplanted cells have to reduce the inflammatory response, inhibit neuronal apoptosis/necrosis, enhance neuronal regeneration, and promote axonal regeneration and remyelination [97]. Cell transplantation augments the neuronal regeneration after spinal cord injury through secretion of paracrine factors, acting as a scaffold for axonal regrowth and replacing the lost neurons or neural progenitor cells [98]. Based on the choice of the cells used for transplantation, cellular based therapy can be broadly classified into pluripotent stem cells, fetal stem cells, progenitor cells, and differentiated cells. Further, the cells can be genetically engineered to enhance the therapeutic functionality of the cells. Various gene therapy approaches have been reported for treating spinal cord injury. Some of the notable genes overexpressed include transcription factors (Ngn2), neurotrophic factors (NT3, BDNF, GDNF, and MNTS1), growth factors (bFGF, HGF), receptor tyrosine kinases (TrKC), and cell adhesion molecule (L1CAM) (Table 7).

## 5. Animal Models and Preclinical/Nonclinical Studies

Various types of animal models such as mouse, rat, dogs, and nonhuman primates, such as marmoset, have been employed to undertake preclinical studies. A recent review after systematic meta-analysis of the animal studies points out that stem cell therapy might offer some hope for cure in spinal cord injury [99]. The pluripotent stem cells like embryonic stem cells (ESCs) and induced pluripotent stem cells (iPSCs) are the type of cells that possess the ability to give rise to all the three germ layers [100, 101]. Recent studies have demonstrated that pluripotent stem cells alone or in combination with other cell types might help in augmenting the recovery from spinal cord injury (Table 1). Bottai et al. directly transplanted mESCs into the lesion immediately after injury and observed that significant function BBB score in the transplanted mice [19]. Nevertheless, there is significant risk of teratoma formation in transplanting the undifferentiated pluripotent cells [102]. Hence the pluripotent stem cells are differentiated into neuronal progenitors, motor neurons, oligodendrocyte progenitor cells, and olfactory ensheathing cells* in vitro* prior to* in vivo* transplantation. Kumagai et al. observed that transplantation of ESCs derived neurospheres in mouse model increased the functional recovery following spinal cord injury [21]. With the help of improved neuronal differentiation protocol Fujimoto et al. showed that transplantation of hiPSCs derived neural epithelial cells resulted in improved axonal regeneration and remyelination [22]. Okano's lab has shown that human iPSCs derived neural cells promote functional recovery after spinal cord injury using nonhuman primate marmoset model [23]. Transplantation enhanced increased axonal regeneration, myelination, and angiogenesis with complete absence of teratomas. An intriguing study by Matsuda et al. has shown that c-transplantation of ESCs with MSCs resulted in no teratoma formation [20]. It has been suggested that MSCs might propel the undifferentiated ESCs to neural cell lineage through mediation of the neurotrophic factors released by them. Additionally, pervious data had revealed that neural cell adhesion molecule L1 is reported to promote survival and axonal growth [103] (Table 7). Transplantation of L1 expressing mESC differentiated into neurons significantly improved the motor function and increased cell survival [24]. Neurogenin 2 (Ngn2) is a transcription factor involved in CNS development [104]. Ngn2 expressing hESCs differentiated neural cells significantly restored the motor functions of the SCI rat model [25]. Combinatorial therapy of hESCs derived neural cells embedded along with collagen scaffold promoted locomotory recovery and prolonged the survival of the graft [26]. Niapour et al. combined hESCs derived neural progenitor cells along with Schwann cells (SCs) to rescue SCI in rat contusion model and observed significant improvement motor functional improvement [27]. The motoneuron progenitor cells (MPCs) [28] and oligodendrocyte progenitor cells (OPCs) [29–31] derived from hESCs have been successfully transplanted in SCI rat models, resulting in significant improvement in functional neuronal regeneration and reduced acute inflammation. Effective promotion of functional recovery and axonal remyelination was observed in transplantation of hESCs derived MPCs along with OPCs [32] and olfactory ensheathing cells (OECs) [33]. Kim et al. reported improved mechanical sensitivity in rat SCI hemisection models after transplantation of mESCs dedifferentiated GABAergic neurons [34].

Mesenchymal stromal cells (MSCs) are a kind of mesodermal stem cells that exhibit plasticity to give rise to cells from all the three germ layers under* in vitro* condition. The ease of availability [105], rapid expansion [106], cryopreservation [107], and subtle immunological complications makes the cells ideal candidates for cellular therapy [108, 109]. Previous preclinical studies have indicated that utilization of MSCs for treatment of spinal cord injury resulted in reduction in demyelination, suppression of neuroinhibitory molecules, and promoting axonal regeneration [110] (Table 2). Transplantation of bone marrow derived MSCs (BMMSCs) derived from rats into SCI rat models revealed slight improvement in neural regeneration with significant restoration of motor functions and attenuation of inflammatory response was found [35–42]. Similar observations were recorded after transplantation of mice BMMSCs into spinal cord injury mouse models [111]. Injection of human BMMSCs [44, 45] and human UCMSCs [46] into the SCI animal models effectively promoted the functional recovery following spinal cord injury. Differentiation of rat [47, 48], canine [49], and human MSCs [112] into neuronal cells prior to transplantation has shown to significantly augment neural regeneration and motor functional recovery with reduction in inflammatory cells. Cotransplantation of r-BMMSCs [77] and h-BMMSCs [113] with Schwann cells (SCs) into SCI rat models resulted in increased axonal remyelination and motor function along with reduced scar formation. Transplantation of genetically modified MSCs expressing growth factors such as bFGF [51] and neurotrophin 3 (NT3) [52, 53] has been shown to improve neuronal functions. Hepatocyte growth factor (HGF) is a paracrine factor secreted by the MSCs. It is a morphogenetic factor that helps in growth and survival of cells [114]. A specific study has observed that transplantation of human BMMSCs expressing HGF in rat hemisection SCI model reduced the glial scar formation by repressing the astrocyte activation and ameliorated the functional recovery of forepaw [54]. Brain-derived neurotrophic factor (BDNF) is a neurotrophic factor encoded by BDNF gene. It has been shown to enhance the neuronal regeneration capabilities in the corticospinal tracks [115]. Grafting of hBMMSCs expressing BDNF in rat SCI model helped in regaining the locomotory function [55]. Glial cell line derived neurotrophic factor (GDNF) is a secretory protein encoded by GDNF gene. GDNF is necessary for normal neuromuscular development [116]. Further, it has been shown that it helps in the survival of motor neurons [117]. Assessment of rat SCI transplanted with rBMMSCs expressing GDNF indicated moderate neural regeneration [56]. MNTS1 is a multineurotrophin that binds and autophosphorylates Trk receptor tyrosine kinases (TrkA/TrkB/TrKC) and p75 neurotrophin receptor (p75 NTR) [118]. Kumagai et al. observed that transplantation of rBMMSCs expressing MNTS1 resulted in suppression of inflammation, reduction in cavity size, and improved neuronal regeneration [57]. Trk family [119] and p75 NTR [120] have been extensively associated with neuronal survival. Improvement in functional motor recovery was observed after transplantation of rBMMSCs expressing TrkC proteins in SCI rat models [58].

The existence of neural progenitor cells (NPCs) was first identified, isolated, and cultured from subventrical zone of the mouse [121, 122]. These NPCs have the ability to differentiate into neurons, astrocytes, and oligodendrocytes under both* in vitro *and* in vivo *conditions. Multiple studies have shown that transplantation of NPCs derived from fetal sources (human/rat/mice) into SCI models has resulted in efficient regeneration of neural structures with function recovery and reduced inflammatory response [59–63] (Table 3). Åkesson et al. observed that human neurospheres obtained from the spinal cord tissue facilitated neuronal regeneration after transplantation into rat spinal cord lesion [64]. The differentiation of NPCs into oligodendrocyte progenitor cells (OPCs) has shown to significantly increase axonal remyelination with better motor and sensory recovery [112]. Further combinatorial studies have indicated that cotransplantation of NPCs with OECs has promoted functional recovery [66]. Hwang et al. reported that transplantation of Olig2 expressing NPCs enhances the locomotory recovery with increase in myelination and reduction in lesion cavity [65]. Genetically modified NSCs expressing TrkC gene along with gelatin sponge scaffold seeded with NT3 helped in bridging the injury site, promoted axonal regeneration, and promoted partial locomotory functional recovery [67].

Olfactory ensheathing cells (OECs) are a class of glial cells that are found in both PNS and CNS [123] (Table 4). Tharion's lab [70] and others [68, 69, 71] have shown that transplantation of rat OECs and mouse OECs into SCI models has shown satisfactory process in the functional recovery and neural tissue restoration. Cotransplantation of OECs with motor neurons [33] yielded significant progress in regeneration capabilities displaying synergistic effect when compared to results obtained from transplantation of OECs with MSCs [72]. Injection of NT3 expressing OECs into rat SCI lesion resulted in neural stimulation and longer survival of the graft with significant increase in motor functional recovery [73].

Schwann cells are the type of glial cells that are associated with myelination of the axonal structures. After SCI, transplantation of Schwann cells has been observed to result in axonal regeneration and remyelination [124] (Table 5). These cells further secrete neurotrophic factors such as nerve growth factor (NGF), brain-derived neurotrophic factors (BDNF), and ciliary neurotrophic factors (CNTF), extracellular matrix proteins that mainly include laminin and collagens, and upregulate cell adhesion molecules like integrins, N-cadherins, N-CAM, L1, and contactins [124–126]. Transplantation of SCs into the SCI lesion has shown to augment the neuronal functional regeneration capabilities along with improved axonal myelination [74–76]. Additionally, cotransplantation of MSCs [77, 78] and NSCs [79] along with SCs has been shown to reduce scar formation and restore the neural functional potential.

## 6. Clinical Trials

**Figure 2:**
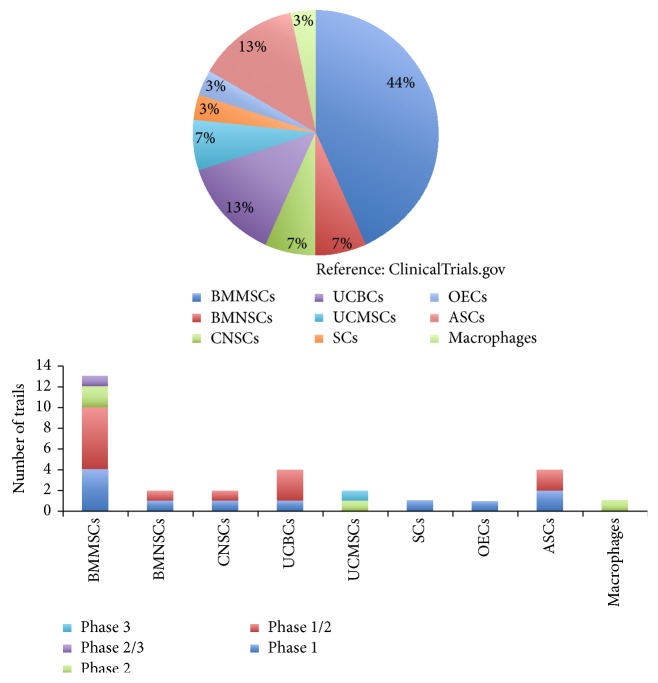
Clinical trials in spinal cord injury using cell therapy. Bone marrow MSCs (BMMSCs), bone marrow mononuclear cells (BMNSCs), central nervous system cells (CNSCs), umbilical cord blood cells (UCBCs), umbilical cord blood MSCs (UCBMSCs), Schwann cells (SCs), olfactory ensheathing cells (OECs), and adipocyte stem cells (ASCs).

The success of the cellular transplantation studies at the preclinical levels resulted in extrapolating a similar therapeutic strategy at the clinical levels (Table 6, Figure 2). In 2009, Geron Corporation was the first to get FDA approval to initiate clinical transplantation of ESCs derived OPCs (GRNOPC1) on spinal cord injury patients [17]. The Phase I clinical trial data did not indicate any improvement in therapeutic potential. However, there are no reported adversities till date following transplantation. In 2011, Geron abruptly ended its clinical trial citing financial limitations. Nevertheless, initiation of the study paved the way for regularizing the following stem cell studies. Unlike the ESCs/iPSCs derived cells, other cell types such as MSCs, NSCs/NPCs, OECs, and SCs exhibit higher safety standards. In a transplantation study involving 171 patients, Huang et al., in 2003, reported functional recovery after transplantation of olfactory ensheathing cells [80]. Further, in 2005, a study by Kang et al. has shown that transplantation of MSCs from human umbilical cord blood into 37-year-old spinal cord injury patient resulted in the functional recovery [81]. Cotransplantation of umbilical cord derived MSCs (UCMSCs) and CD34+ HSCs (UCHSCs) on a 29-year-old L1 SCI American Spinal Injury Association (ASIA) Scale Type A patient resulted in noted recovery of muscle, bowl, and sexual function [82]. There were no reported adversities during the study and ASIA Scale was reduced to Type D. A critical study including 64 patients by Kishk et al. observed limited progress after autologous intrathecal transplantation of BMMSCs [83]. The ASIA Scales of the patients were rated down from Type A to Type B. However some of the patients exhibited complications such as cell induced spasticity, neuropathic pain, and development of encephalomyelitis. Bhanot et al. reported that transplantation of autologous MSCs on 13 SCI patients (ASIA Scale Type A) at the site of the lesion resulted in sensory functional recovery in 2 patients and motor function recovery in only one patient [84]. In a similar study involving 10 SCI patients, transplantation of autologous BMMSCs showed significant improvement in motor/sensory functional recovery in 6 patients [86]. Moreover, MRI studies indicated neurogenesis and decrease in the cavity size, and electrophysiological analysis indicated improved functional potential. There were no reported complications. Karamouzian et al. demonstrated that transplantation of autologous BMMSCs via lumbar puncture into CSF in 11 SCI patients resulted in borderline functional recovery in 5 patients [85]. In a study involving Schwann cells (SCs), Saberi et al. reported that transplantation of autologous SCs into 4 SCI patients resulted in marginal functional improvement in only one patient [87]. No complications were reported in any of the patients. In a follow-up study involving 33 patients, 16 patients ASIA Scale Type A and 17 patients ASIA Scale Type B, upon transplantation of SCs, up to 6 patients had shown progress in bladder and bowl control [88]. Some of the patients had exhibited some function recovery, which was not very significant. In another study, Zhou et al. described that injection of the autologous SCs in 6 SCI patients led to moderate improvement in all of the treated patients in terms of anatomical, motor, and sensory functions after follow-up of 5 years [89].

## 7. Limitations and Ethical Concerns

Although the various strategies are employed for the treatment of spinal cord injury, until now there is no proper cure that is safe and effective for spinal cord injury patients [17]. Although cell based interventions hold a great promise in the treatment of spinal cord injury, it is at its nascent stages; still a lot of multicentric studies are required as there are variations in treatment regime from one clinical setting to another. Further questions like type of cell to be used, site of transplantation, dosage, and number of cells are not properly standardized. The mechanisms governing injury and regeneration are not properly understood. There are very limited preclinical and clinical studies reported at present. Due to poor regulations, there are a lot of unethical practices associated with stem cell transplantation. The 2003 transplantation study reported by Huang et al. was received with skepticism and ethical concerns [127–129]. Still a lot of research has to be carried out at preclinical levels, which include screening using small animal models (rats and mice), large animal models (cats, dogs, rabbit, and primates), and clinical levels to optimize various parameters until it becomes standard of care [130]. The propensity of tumor development after transplantation is significantly higher in case of pluripotent stem cells derived neural cells. Transplantation of hESCs derived NPCs into spinal cord of SCID mice has been shown to result in teratoma [131]. Hence pluripotent stem cell derived neural cells sources have to be subjected to rigorous selection before transplantation.

## 8. Conclusion

Currently, very few places have spinal cord injury registry. Hence maintenance of spinal cord injury registry has to be promoted in order to properly take care and evaluate spinal cord injury patients. Currently, there is no proper cure for SCI therapy. The current preclinical and clinical data indicate that cell therapy may hold key to future regenerative medical applications. There are lots of animal models and clinical data that are incompletely evaluated leading towards utter confusion in the field. The researchers working in spinal cord injury have to communicate, coordinate, and conduct multicentric clinical trials in order to generate some meaning of full consensus with respect to cell therapy in the field of spinal cord injury. Moreover, cellular therapy can be combined with genetic modification or small molecules as a combinatorial approach to deliver the cure. Our current goal should be to regain some functional recovery and neuronal regeneration. We cannot expect the injured person to get up and start walking. Still a lot of questions like the type of cell, number of cells, and region of transplant have to be answered. At present, it is difficult to ascertain the fate of the cells. Further,* in vivo* tracking technology of the cells is at its nascent stages. Hence, development of* in vivo* tracking has to be given due importance.

## Figures and Tables

**Table 1:** Preclinical spinal cord injury trials using induced pluripotent stem cells (iPSCs)/embryonic stem cells (ESCs).

Transplanted cell type	SCI model	Neuronal regeneration	Functional recovery	Inflammation repression	Reference
mESCs	Mice, T8, contusion	—	Yes	Yes	[19]
mESCs + mMSCs	Mice, T9-10, contusion	Yes	—	—	[20]
mESCs-neurosphere	Mice, T10, contusion	Yes	Yes	—	[21]
hiPSCs-neuroepithelial cells	Mice, T10, contusion	Yes	Yes	—	[22]
hiPSCs-neural cells	Marmoset, C5, contusion	Yes	Yes	—	[23]
L1-mESCs-neural cells	Mice, T9, compression	Yes	Yes	Yes	[24]
Ngn2-hESCs	Rats, T9, compression	Yes	Yes	—	[25]
hECSs-NPCs	Rats, T10, hemisection	Yes	Yes	—	[26]
hESCs-NPCs + SCs	Rat, T9, contusion	Yes	Yes	—	[27]
hESCs-MPCs	Rat, C5-C6, contusion	Yes	Yes	—	[28]
hESCs-OPCs	Rat, T10, contusion	Yes	Yes	—	[29]
hESCs-OPCs	Rat, T9, contusion	Yes	Yes	—	[30]
hESCs-OPCs	Rat, C5, contusion	Yes	Yes	Yes	[31]
hESCs-MPCs/OPCs	Rat, T8, complete transection	Yes	Yes	—	[32]
hESCs-MPCs + OECs	Rat, T9, complete transection	Yes	Yes	—	[33]
mESCs-GABAergic	Rat, T13, lateral hemisection	Yes	Yes	—	[34]

Mouse embryonic stem cells (mESCs); human embryonic stem cells (hESCs); human induced pluripotent stem cells (hiPSCs); neural progenitor cells (NPCs); Schwann cells (SCs); neurogenin 2 (Ngn2); motoneuron progenitor cells (MPCs); oligodendrocyte progenitor cells (OPCs); olfactory ensheathing cells (OECs).

**Table 2:** Preclinical spinal cord injury trials using mesenchymal stromal cells (MSCs).

Transplanted cell type	SCI model	Neuronal regeneration	Functional recovery	Inflammation repression	Reference
r-BMMSCs	Rat, compression/contusion	Partial	Yes	Yes	[35–42]
m-BMMSCs	Mice, T9, compression	Partial	Yes	—	[43]
h-BMMSCs	Rat, T8-T9, complete transection	Yes	Yes	Yes	[44, 45]
h-UCMSCs	Dog, L2-3, compression	Yes	Yes	—	[46]
r-BMMSCs-NPCs	Rat, T8-T9, contusion/compression	Yes	Yes	—	[47, 48]
Canine-aMSCs-NPCs	Dogs, L2-3, compression	Yes	Yes	Yes	[49]
h-BMMSCs-NPCs	Rats, T9, contusion	Yes	Yes	—	[50]
r-BMMSCs-bFGF	Rats, T9, contusion	Yes	Yes	—	[51]
r-BMMSCs-NT3	Rats, T9, ethidium bromide induced demyelination	Yes	Yes	—	[52]
r-BMMSCs-NT3	Rats, T9, contusion	Yes	Yes	—	[53]
h-BMMSCs-HGF	Rats, C4, hemisection	Yes	Yes	Yes	[54]
h-BMMSCs-BDNF	Rats, T9, transection	Yes	Yes	—	[55]
r-BMMSCs-GDNF	Rats, T9, contusion	Partial	—	—	[56]
r-BMMSCs-MNTS1	Rats, T8, contusion	Yes	Yes	Yes	[57]
r-BMMSCs-TrkC	Rats, T10, complete transection	Yes	Yes	—	[58]

Rat bone marrow MSCs (rBMMSCs); human umbilical cord MSCs (hUCMSCs); basic fibroblast growth factors (bFGF); neurotrophin 3 (NT3); hepatocyte growth factor (HGF); brain derived neurotrophic factor (BDNF); glial cell line derived neurotrophic factor (GDNF).

**Table 3:** Preclinical spinal cord injury trials using neural stem cells (NSCs)/neural progenitor cells (NPCs).

Transplanted cell type	SCI model	Neuronal regeneration	Functional recovery	Inflammation repression	Reference
Fetal-mNSCs	Mice, T10, contusion	Yes	Yes	—	[59]
Fetal-rNSCs	Rats, C4, dorsal hemisection	No	Partial	—	[60]
Fetal-hNSCs	Rats/mice, contusion/avulsion	Yes	Yes	—	[61–63]
Spinal cord-hNPCs	Rats, T8, compression	Yes	—	—	[64]
Fetal-hNPCs-OPCs	Rats, T8, compression	Yes	Yes	—	[50]
Fetal-hNSCs-Olig2	Rats, T9-10, contusion	Yes	Yes	—	[65]
Fetal-rNSCs + OECs	Rats, T8, compression	Yes	Yes	—	[66]
rNSCs-TrkC + NT-3	Rats, T10, transection	Yes	Yes	—	[67]

Oligodendrocyte progenitor cells (OPCs); olfactory ensheathing cells (OECs).

**Table 4:** Preclinical spinal cord injury trials using olfactory ensheathing cells (OECs).

Transplanted cell type	SCI model	Neuronal regeneration	Functional recovery	Inflammation repression	Reference
rOECs	Rats, contusion/compression/ Transection/hemisection	Yes	Yes	—	[68–71]
rOECs + motor neurons	Rat, T9, transection	Yes	Yes	—	[33]
rOECs + MSCs	Rat, T8, compression	Yes	Partial	—	[72]
rOECs-NT3	Rat, T8, compression	—	Partial	—	[73]

Neurotrophin 3 (NT3).

**Table 5:** Preclinical spinal cord injury trials using Schwann cells (SCs).

Transplanted cell type	SCI model	Neuronal regeneration	Functional recovery	Inflammation repression	Reference
rSCs	Rats, contusion/compression/hemisection	Yes	Yes	—	[74–76]
rSCs + MSCs	Rat, contusion/4 mm spinal cord removal	Yes	Yes	—	[77, 78]
rSC + NSCs	Rat, T8-9, transection	Yes	Partial	—	[79]

**Table 6:** Clinical spinal cord injury trials using cell therapy.

Transplanted cell type	SCI model	Safety	Neuronal Regeneration and functional recovery	Inflammation repression	Reference
ESCs-OPCs	Phase 1, ASIA Scale Type A	Yes	—	—	[17]
OECs	171 Patients	Yes	Yes	—	[80]
UCMSCs	1, T11-12, ASIA Scale Type A	Yes	Yes	—	[81]
UCMSCs + CD34^+^ HSCs	L1, ASIA Scale Type A	Yes	Yes	—	[82]
BMMSCs	ASIA Scale Type A	Partial (spasticity/neuropathic pain)	Yes	Partial	[83]
BMMSCs	ASIA Scale Type A/B/C	Yes	Yes	Partial	[84–86]
SCs	ASIA Scale Type A/B/C	Partial	Yes	—	[87–89]

**Table 7:** Genes used for engineering cells.

Genes	Carrier	Reference
L1	Electroporation	[24]
Ngn2	Lentivirus	[25]
Olig2	Retrovirus	[65]
bFGF	Transfection	[51]
HGF	Lentivirus	[54]
NT3	Adenovirus	[52]
BDNF	Adenovirus	[55]
GDNF	Retrovirus	[56]
MNTS1	Lentivirus	[57]
TrkC	Adenovirus	[58]

## References

[1] Yip P. K., Malaspina A. (2012). Spinal cord trauma and the molecular point of no return. *Molecular Neurodegeneration*.

[2] Cripps R. A., Lee B. B., Wing P., Weerts E., MacKay J., Brown D. (2011). A global map for traumatic spinal cord injury epidemiology: towards a living data repository for injury prevention. *Spinal Cord*.

[3] Sebastià-Alcácer V., Alcanyis-Alberola M., Giner-Pascual M., Gomez-Pajares F. (2014). Are the characteristics of the patient with a spinal cord injury changing?. *Spinal Cord*.

[4] Wyndaele M., Wyndaele J.-J. (2006). Incidence, prevalence and epidemiology of spinal cord injury: what learns a worldwide literature survey?. *Spinal Cord*.

[5] Cui X., Chen L., Ren Y. (2013). Genetic modification of mesenchymal stem cells in spinal cord injury repair strategies. *BioScience Trends*.

[6] Albert T., Ravaud J.-F., Papa A. (2005). Rehabilitation of spinal cord injury in France: a nationwide multicentre study of incidence and regional disparities. *Spinal Cord*.

[7] Pickett W., Simpson K., Walker J., Brison R. J. (2003). Traumatic spinal cord injury in Ontario, Canada. *The Journal of Trauma—Injury, Infection and Critical Care*.

[8] New P. W., Rawicki H. B., Bailey M. J. (2002). Nontraumatic spinal cord injury: demographic characteristics and complications. *Archives of Physical Medicine and Rehabilitation*.

[9] Thuret S., Moon L. D. F., Gage F. H. (2006). Therapeutic interventions after spinal cord injury. *Nature Reviews Neuroscience*.

[10] McDonald J. W., Sadowsky C. (2002). Spinal-cord injury. *The Lancet*.

[11] Tator C. H. (1995). Update on the pathophysiology and pathology of acute spinal cord injury. *Brain Pathology*.

[12] Bauchet L., Lonjon N., Perrin F.-E., Gilbert C., Privat A., Fattal C. (2009). Strategies for spinal cord repair after injury: a review of the literature and information. *Annals of Physical and Rehabilitation Medicine*.

[13] Kakulas B. A. (1999). A review of the neuropathology of human spinal cord injury with emphasis on special features. *The Journal of Spinal Cord Medicine*.

[14] Norenberg M. D., Smith J., Marcillo A. (2004). The pathology of human spinal cord injury: defining the problems. *Journal of Neurotrauma*.

[15] Rowland J. W., Hawryluk G. W. J., Kwon B., Fehlings M. G. (2008). Current status of acute spinal cord injury pathophysiology and emerging therapies: promise on the horizon. *Neurosurgical Focus*.

[16] Deumens R., Koopmans G. C., Honig W. M. M. (2006). Chronically injured corticospinal axons do not cross large spinal lesion gaps after a multifactorial transplantation strategy using olfactory ensheathing cell/olfactory nerve fibroblast-biomatrix bridges. *Journal of Neuroscience Research*.

[17] Li J., Lepski G. (2013). Cell transplantation for spinal cord injury: a systematic review. *BioMed Research International*.

[18] Nakamura M., Okano H. (2013). Cell transplantation therapies for spinal cord injury focusing on induced pluripotent stem cells. *Cell Research*.

[19] Bottai D., Cigognini D., Madaschi L. (2010). Embryonic stem cells promote motor recovery and affect inflammatory cell infiltration in spinal cord injured mice. *Experimental Neurology*.

[20] Matsuda R., Yoshikawa M., Kimura H. (2009). Cotransplantation of mouse embryonic stem cells and bone marrow stromal cells following spinal cord injury suppresses tumor development. *Cell Transplantation*.

[21] Kumagai G., Okada Y., Yamane J. (2009). Roles of ES cell-derived gliogenic neural stem/progenitor cells in functional recovery after spinal cord injury. *PLoS ONE*.

[22] Fujimoto Y., Abematsu M., Falk A. (2012). Treatment of a mouse model of spinal cord injury by transplantation of human induced pluripotent stem cell-derived long-term self-renewing neuroepithelial-like stem cells. *Stem Cells*.

[23] Kobayashi Y., Okada Y., Itakura G. (2012). Pre-evaluated safe human iPSC-derived neural stem cells promote functional recovery after spinal cord injury in common marmoset without tumorigenicity. *PLoS ONE*.

[24] Cui Y.-F., Xu J.-C., Hargus G., Jakovcevski I., Schachner M., Bernreuther C. (2011). Embryonic stem cell-derived L1 overexpressing neural aggregates enhance recovery after spinal cord injury in mice. *PLoS ONE*.

[25] Perrin F. E., Boniface G., Serguera C. (2010). Grafted human embryonic progenitors expressing neurogenin-2 stimulate axonal sprouting and improve motor recovery after severe spinal cord injury. *PLoS ONE*.

[26] Hatami M., Mehrjardi N. Z., Kiani S. (2009). Human embryonic stem cell-derived neural precursor transplants in collagen scaffolds promote recovery in injured rat spinal cord. *Cytotherapy*.

[27] Niapour A., Karamali F., Nemati S. (2012). Cotransplantation of human embryonic stem cell-derived neural progenitors and Schwann cells in a rat spinal cord contusion injury model elicits a distinct neurogenesis and functional recovery. *Cell Transplantation*.

[28] Rossi S. L., Nistor G., Wyatt T. (2010). Histological and functional benefit following transplantation of motor neuron progenitors to the injured rat spinal cord. *PLoS ONE*.

[29] Keirstead H. S., Nistor G., Bernal G. (2005). Human embryonic stem cell-derived oligodendrocyte progenitor cell transplants remyelinate and restore locomotion after spinal cord injury. *The Journal of Neuroscience*.

[30] Kerr C. L., Letzen B. S., Hill C. M. (2010). Efficient differentiation of human embryonic stem cells into oligodendrocyte progenitors for application in a rat contusion model of spinal cord injury. *International Journal of Neuroscience*.

[31] Sharp J., Frame J., Siegenthaler M., Nistor G., Keirstead H. S. (2010). Human embryonic stem cell-derived oligodendrocyte progenitor cell transplants improve recovery after cervical spinal cord injury. *Stem Cells*.

[32] Erceg S., Ronaghi M., Oria M. (2010). Transplanted oligodendrocytes and motoneuron progenitors generated from human embryonic stem cells promote locomotor recovery after spinal cord transection. *Stem Cells*.

[33] Salehi M., Pasbakhsh P., Soleimani M. (2009). Repair of spinal cord injury by co-transplantation of embryonic stem cell-derived motor neuron and olfactory ensheathing cell. *Iranian Biomedical Journal*.

[34] Kim D.-S., Jung Jung S. E., Nam T. S. (2010). Transplantation of GABAergic neurons from ESCs attenuates tactile hypersensitivity following spinal cord injury. *Stem Cells*.

[35] Abrams M. B., Dominguez C., Pernold K. (2009). Multipotent mesenchymal stromal cells attenuate chronic inflammation and injury-induced sensitivity to mechanical stimuli in experimental spinal cord injury. *Restorative Neurology and Neuroscience*.

[36] Gu W., Zhang F., Xue Q., Ma Z., Lu P., Yu B. (2010). Transplantation of bone marrow mesenchymal stem cells reduces lesion volume and induces axonal regrowth of injured spinal cord. *Neuropathology*.

[37] Kang E.-S., Ha K.-Y., Kim Y.-H. (2012). Fate of transplanted bone marrow derived mesenchymal stem cells following spinal cord injury in rats by transplantation routes. *Journal of Korean Medical Science*.

[38] Karaoz E., KabataS S., Duruksu G. (2012). Reduction of lesion in injured rat spinal cord and partial functional recovery of motility after bone marrow derived mesenchymal stem cell transplantation. *Turkish Neurosurgery*.

[39] Mothe A. J., Bozkurt G., Catapano J. (2011). Intrathecal transplantation of stem cells by lumbar puncture for thoracic spinal cord injury in the rat. *Spinal Cord*.

[40] Nakajima H., Uchida K., Guerrero A. R. (2012). Transplantation of mesenchymal stem cells promotes an alternative pathway of macrophage activation and functional recovery after spinal cord injury. *Journal of Neurotrauma*.

[41] Osaka M., Honmou O., Murakami T. (2010). Intravenous administration of mesenchymal stem cells derived from bone marrow after contusive spinal cord injury improves functional outcome. *Brain Research*.

[42] Park W. B., Kim S. Y., Lee S. H., Kim H.-W., Park J.-S., Hyun J. K. (2010). The effect of mesenchymal stem cell transplantation on the recovery of bladder and hindlimb function after spinal cord contusion in rats. *BMC Neuroscience*.

[43] Boido M., Garbossa D., Fontanella M., Ducati A., Vercelli A. (2014). Mesenchymal stem cell transplantation reduces glial cyst and improves functional outcome after spinal cord compression. *World Neurosurgery*.

[44] Kang K. N., Kim D. Y., Yoon S. M. (2012). Tissue engineered regeneration of completely transected spinal cord using human mesenchymal stem cells. *Biomaterials*.

[45] Zeng X., Zeng Y.-S., Ma Y.-H. (2011). Bone marrow mesenchymal stem cells in a three-dimensional gelatin sponge scaffold attenuate inflammation, promote angiogenesis, and reduce cavity formation in experimental spinal cord injury. *Cell Transplantation*.

[46] Lee J.-H., Chung W.-H., Kang E.-H. (2011). Schwann cell-like remyelination following transplantation of human umbilical cord blood (hUCB)-derived mesenchymal stem cells in dogs with acute spinal cord injury. *Journal of the Neurological Sciences*.

[47] Cho S.-R., Kim Y. R., Kang H.-S. (2009). Functional recovery after the transplantation of neurally differentiated mesenchymal stem cells derived from bone barrow in a rat model of spinal cord injury. *Cell Transplantation*.

[48] Pedram M. S., Dehghan M. M., Soleimani M., Sharifi D., Marjanmehr S. H., Nasiri Z. (2010). Transplantation of a combination of autologous neural differentiated and undifferentiated mesenchymal stem cells into injured spinal cord of rats. *Spinal Cord*.

[49] Park S.-S., Lee Y. J., Lee S. H. (2012). Functional recovery after spinal cord injury in dogs treated with a combination of Matrigel and neural-induced adipose-derived mesenchymal Stem cells. *Cytotherapy*.

[50] Alexanian A. R., Fehlings M. G., Zhang Z., Maiman D. J. (2011). Transplanted neurally modified bone marrow-derived mesenchymal stem cells promote tissue protection and locomotor recovery in spinal cord injured rats. *Neurorehabilitation and Neural Repair*.

[51] Liu W.-G., Wang Z.-Y., Huang Z.-S. (2011). Bone marrow-derived mesenchymal stem cells expressing the bFGF transgene promote axon regeneration and functional recovery after spinal cord injury in rats. *Neurological Research*.

[52] Zhang Y.-J., Zhang W., Lin C.-G. (2012). Neurotrophin-3 gene modified mesenchymal stem cells promote remyelination and functional recovery in the demyelinated spinal cord of rats. *Journal of the Neurological Sciences*.

[53] Shang A.-J., Hong S.-Q., Xu Q. (2011). NT-3-secreting human umbilical cord mesenchymal stromal cell transplantation for the treatment of acute spinal cord injury in rats. *Brain Research*.

[54] Jeong S. R., Kwon M. J., Lee H. G. (2012). Hepatocyte growth factor reduces astrocytic scar formation and promotes axonal growth beyond glial scars after spinal cord injury. *Experimental Neurology*.

[55] Sasaki M., Radtke C., Tan A. M. (2009). BDNF-hypersecreting human mesenchymal stem cells promote functional recovery, axonal sprouting, and protection of corticospinal neurons after spinal cord injury. *The Journal of Neuroscience*.

[56] Rooney G. E., McMahon S. S., Ritter T. (2009). Neurotrophic factor-expressing mesenchymal stem cells survive transplantation into the contused spinal cord without differentiating into neural cells. *Tissue Engineering—Part A*.

[57] Kumagai G., Tsoulfas P., Toh S., McNiece I., Bramlett H. M., Dietrich W. D. (2013). Genetically modified mesenchymal stem cells (MSCs) promote axonal regeneration and prevent hypersensitivity after spinal cord injury. *Experimental Neurology*.

[58] Ding Y., Yan Q., Ruan J.-W. (2013). Electroacupuncture promotes the differentiation of transplanted bone marrow mesenchymal stem cells overexpressing TrkC into neuron-like cells in transected spinal cord of rats. *Cell Transplantation*.

[59] Yasuda A., Tsuji O., Shibata S. (2011). Significance of remyelination by neural stem/progenitor cells transplanted into the injured spinal cord. *Stem Cells (Dayton, Ohio)*.

[60] Webber D. J., Bradbury E. J., McMahon S. B., Minger S. L. (2007). Transplanted neural progenitor cells survive and differentiate but achieve limited functional recovery in the lesioned adult rat spinal cord. *Regenerative Medicine*.

[61] Salazar D. L., Uchida N., Hamers F. P. T., Cummings B. J., Anderson A. J. (2010). Human neural stem cells differentiate and promote locomotor recovery in an early chronic spinal cord injury NOD-scid mouse model. *PLoS ONE*.

[62] Tarasenko Y. I., Gao J., Nie L. (2007). Human fetal neural stem cells grafted into contusion-injured rat spinal cords improve behavior. *Journal of Neuroscience Research*.

[63] Yan J., Xu L., Welsh A. M. (2007). Extensive neuronal differentiation of human neural stem cell grafts in adult rat spinal cord. *PLoS Medicine*.

[64] Åkesson E., Piao J.-H., Samuelsson E.-B. (2007). Long-term culture and neuronal survival after intraspinal transplantation of human spinal cord-derived neurospheres. *Physiology & Behavior*.

[65] Hwang D. H., Kim B. G., Kim E. J. (2009). Transplantation of human neural stem cells transduced with Olig2 transcription factor improves locomotor recovery and enhances myelination in the white matter of rat spinal cord following contusive injury. *BMC Neuroscience*.

[66] Wang G., Ao Q., Gong K., Zuo H., Gong Y., Zhang X. (2010). Synergistic effect of neural stem cells and olfactory ensheathing cells on repair of adult rat spinal cord injury. *Cell Transplantation*.

[67] Du B.-L., Zeng X., Ma Y.-H. (2014). Graft of the gelatin sponge scaffold containing genetically-modified neural stem cells promotes cell differentiation, axon regeneration, and functional recovery in rat with spinal cord transection. *Journal of Biomedical Materials Research A*.

[68] Liu K.-J., Xu J., Yang C.-Y. (2010). Analysis of olfactory ensheathing glia transplantation-induced repair of spinal cord injury by electrophysiological, behavioral, and histochemical methods in rats. *Journal of Molecular Neuroscience*.

[69] Stamegna J. C., Felix M. S., Roux-Peyronnet J. (2011). Nasal OEC transplantation promotes respiratory recovery in a subchronic rat model of cervical spinal cord contusion. *Experimental Neurology*.

[70] Tharion G., Indirani K., Durai M. (2011). Motor recovery following olfactory ensheathing cell transplantation in rats with spinal cord injury. *Neurology India*.

[71] Ziegler M. D., Hsu D., Takeoka A. (2011). Further evidence of olfactory ensheathing glia facilitating axonal regeneration after a complete spinal cord transection. *Experimental Neurology*.

[72] Amemori T., Jendelová P., Růžičková K., Arboleda D., Syková E. (2010). Co-transplantation of olfactory ensheathing glia and mesenchymal stromal cells does not have synergistic effects after spinal cord injury in the rat. *Cytotherapy*.

[73] Ma Y.-H., Zhang Y., Cao L. (2010). Effect of neurotrophin-3 genetically modified olfactory ensheathing cells transplantation on spinal cord injury. *Cell Transplantation*.

[74] Agudo M., Woodhoo A., Webber D., Mirsky R., Jessen K. R., McMahon S. B. (2008). Schwann cell precursors transplanted into the injured spinal cord multiply, integrate and are permissive for axon growth. *Glia*.

[75] Biernaskie J., Sparling J. S., Liu J. (2007). Skin-derived precursors generate myelinating Schwann cells that promote remyelination and functional recovery after contusion spinal cord injury. *The Journal of Neuroscience*.

[76] Patel V., Joseph G., Patel A. (2010). Suspension matrices for improved Schwann-cell survival after implantation into the injured rat spinal cord. *Journal of Neurotrauma*.

[77] Ban D.-X., Ning G.-Z., Feng S.-Q. (2011). Combination of activated Schwann cells with bone mesenchymal stem cells: the best cell strategy for repair after spinal cord injury in rats. *Regenerative Medicine*.

[78] Fouad K., Schnell L., Bunge M. B., Schwab M. E., Liebscher T., Pearse D. D. (2005). Combining Schwann cell bridges and olfactory-ensheathing glia grafts with chondroitinase promotes locomotor recovery after complete transection of the spinal cord. *Journal of Neuroscience*.

[79] Olson H. E., Rooney G. E., Gross L. (2009). Neural stem cell- and schwann cell-loaded biodegradable polymer scaffolds support axonal regeneration in the transected spinal cord. *Tissue Engineering—Part A*.

[80] Huang H., Chen L., Wang H. (2003). Influence of patients' age on functional recovery after transplantation of olfactory ensheathing cells into injured spinal cord injury. *Chinese Medical Journal*.

[81] Kang K.-S., Kim S. W., Oh Y. H. (2005). A 37-year-old spinal cord-injured female patient, transplanted of multipotent stem cells from human UC blood, with improved sensory perception and mobility, both functionally and morphologically: a case study. *Cytotherapy*.

[82] Ichim T. E., Solano F., Lara F. (2010). Feasibility of combination allogeneic stem cell therapy for spinal cord injury: a case report. *International Archives of Medicine*.

[83] Kishk N. A., Gabr H., Hamdy S. (2010). Case control series of intrathecal autologous bone marrow mesenchymal stem cell therapy for chronic spinal cord injury. *Neurorehabilitation and Neural Repair*.

[84] Bhanot Y., Rao S., Ghosh D., Balaraju S., Radhika C. R., Kumar K. V. S. (2011). Autologous mesenchymal stem cells in chronic spinal cord injury. *British Journal of Neurosurgery*.

[85] Karamouzian S., Nematollahi-Mahani S. N., Nakhaee N., Eskandary H. (2012). Clinical safety and primary efficacy of bone marrow mesenchymal cell transplantation in subacute spinal cord injured patients. *Clinical Neurology and Neurosurgery*.

[86] Park J. H., Kim D. Y., Sung I. Y. (2012). Long-term results of spinal cord injury therapy using mesenchymal stem cells derived from bone marrow in humans. *Neurosurgery*.

[87] Saberi H., Moshayedi P., Aghayan H.-R. (2008). Treatment of chronic thoracic spinal cord injury patients with autologous Schwann cell transplantation: an interim report on safety considerations and possible outcomes. *Neuroscience Letters*.

[88] Saberi H., Firouzi M., Habibi Z. (2011). Safety of intramedullary Schwann cell transplantation for postrehabilitation spinal cord injuries: 2-year follow-up of 33 cases. *Journal of Neurosurgery: Spine*.

[89] Zhou X.-H., Ning G.-Z., Feng S.-Q. (2012). Transplantation of autologous activated schwann cells in the treatment of spinal cord injury: six cases, more than five years of follow-up. *Cell Transplantation*.

[90] Nesic O., Svrakic N. M., Xu G.-Y. (2002). DNA microarray analysis of the contused spinal cord: effect of NMDA receptor inhibition. *Journal of Neuroscience Research*.

[91] Pan J. Z., Ni L., Sodhi A., Aguanno A., Young W., Hart R. P. (2002). Cytokine activity contributes to induction of inflammatory cytokine mRNAs in spinal cord following contusion. *Journal of Neuroscience Research*.

[92] Bareyre F. M., Schwab M. E. (2003). Inflammation, degeneration and regeneration in the injured spinal cord: insights from DNA microarrays. *Trends in Neurosciences*.

[93] Fan M., Mi R., Yew D. T., Chan W. Y. (2001). Analysis of gene expression following sciatic nerve crush and spinal cord hemisection in the mouse by microarray expression profiling. *Cellular and Molecular Neurobiology*.

[94] di Giovanni S., Knoblach S. M., Brandoli C., Aden S. A., Hoffman E. P., Faden A. I. (2003). Gene profiling in spinal cord injury shows role of cell cycle in neuronal death. *Annals of Neurology*.

[95] Song G., Cechvala C., Resnick D. K., Dempsey R. J., Raghavendra Rao V. L. (2001). GeneChip analysis after acute spinal cord injury in rat. *Journal of Neurochemistry*.

[96] Carmel J. B., Galante A., Soteropoulos P. (2001). Gene expression profiling of acute spinal cord injury reveals spreading inflammatory signals and neuron loss. *Physiol Genomics*.

[97] Garbossa D., Boido M., Fontanella M., Fronda C., Ducati A., Vercelli A. (2012). Recent therapeutic strategies for spinal cord injury treatment: possible role of stem cells. *Neurosurgical Review*.

[98] Pearse D. D., Bunge M. B. (2006). Designing cell- and gene-based regeneration strategies to repair the injured spinal cord. *Journal of Neurotrauma*.

[99] Antonic A., Sena E. S., Lees J. S. (2013). Stem cell transplantation in traumatic spinal cord injury: a systematic review and meta-analysis of animal studies. *PLoS Biology*.

[100] Miura K., Okada Y., Aoi T. (2009). Variation in the safety of induced pluripotent stem cell lines. *Nature Biotechnology*.

[101] Puri M. C., Nagy A. (2012). Concise review: embryonic stem cells versus induced pluripotent stem cells: the game is on. *Stem Cells*.

[102] Tsuji O., Miura K., Fujiyoshi K., Momoshima S., Nakamura M., Okano H. (2011). Cell therapy for spinal cord injury by neural stem/progenitor cells derived from iPS/ES cells. *Neurotherapeutics*.

[103] Chen J., Bernreuther C., Dihné M., Schachner M. (2005). Cell adhesion molecule L1-transfected embryonic stem cells with enhanced survival support regrowth of corticospinal tract axons in mice after spinal cord injury. *Journal of Neurotrauma*.

[104] Galichet C., Guillemot F., Parras C. M. (2008). Neurogenin 2 has an essential role in development of the dentate gyrus. *Development*.

[105] Zhang X., Hirai M., Cantero S. (2011). Isolation and characterization of mesenchymal stem cells from human umbilical cord blood: reevaluation of critical factors for successful isolation and high ability to proliferate and differentiate to chondrocytes as compared to mesenchymal stem cells from bone marrow and adipose tissue. *Journal of Cellular Biochemistry*.

[106] Sekiya I., Larson B. L., Smith J. R., Pochampally R., Cui J.-G., Prockop D. J. (2002). Expansion of human adult stem cells from bone marrow stroma: conditions that maximize the yields of early progenitors and evaluate their quality. *Stem Cells*.

[107] Lee M. W., Yang M. S., Park J. S., Kim H. C., Kim Y. J., Choi J. (2005). Isolation of mesenchymal stem cells from cryopreserved human umbilical cord blood. *International Journal of Hematology*.

[108] Carrade D. D., Affolter V. K., Outerbridge C. A. (2011). Intradermal injections of equine allogeneic umbilical cord-derived mesenchymal stem cells are well tolerated and do not elicit immediate or delayed hypersensitivity reactions. *Cytotherapy*.

[109] Krampera M., Glennie S., Dyson J. (2003). Bone marrow mesenchymal stem cells inhibit the response of naive and memory antigen-specific T cells to their cognate peptide. *Blood*.

[110] Malgieri A., Kantzari E., Patrizi M. P., Gambardella S. (2010). Bone marrow and umbilical cord blood human mesenchymal stem cells: state of the art. *International Journal of Clinical and Experimental Medicine*.

[111] Boido M., Garbossa D., Fontanella M., Ducati A., Vercelli A. (2014). Mesenchymal stem cell transplantation reduces glial cyst and improves functional outcome after spinal cord compression. *World Neurosurgery*.

[112] Alexanian A. R., Svendsen C. N., Crowe M. J., Kurpad S. N. (2010). Transplantation of human glial-restricted neural precursors into injured spinal cord promotes functional and sensory recovery without causing allodynia. *Cytotherapy*.

[113] Guo Y.-W., Ke Y.-Q., Li M. (2011). Human umbilical cord-derived schwann-like cell transplantation combined with neurotrophin-3 administration in dyskinesia of rats with spinal cord injury. *Neurochemical Research*.

[114] Sulpice E., Ding S., Muscatelli-Groux B. (2009). Cross-talk between the VEGF-A and HGF signalling pathways in endothelial cells. *Biology of the Cell*.

[115] Hiebert G. W., Khodarahmi K., McGraw J., Steeves J. D., Tetzlaff W. (2002). Brain-derived neurotrophic factor applied to the motor cortex promotes sprouting of corticospinal fibers but not regeneration into a peripheral nerve transplant. *Journal of Neuroscience Research*.

[116] Wang C.-Y., Yang F., He X.-P. (2002). Regulation of neuromuscular synapse development by glial cell line-derived neurotrophic factor and neurturin. *The Journal of Biological Chemistry*.

[117] Acsadi G., Lewis R. A., Shy M. E. (2002). Increased survival and function of SOD1 mice after Glial cell-derived neurotrophic factor gene therapy. *Human Gene Therapy*.

[118] Urfer R., Tsoulfas P., Soppet D., Escandón E., Parada L. F., Presta L. G. (1994). The binding epitopes of neurotrophin-3 to its receptors trkC and gp75 and the design of a multifunctional human neurotrophin. *The EMBO Journal*.

[119] Huang E. J., Reichardt L. F. (2001). Neurotrophins: roles in neuronal development and function. *Annual Review of Neuroscience*.

[120] Lee K.-F., Davies A. M., Jaenisch R. (1994). p75-deficient embryonic dorsal root sensory and neonatal sympathetic neurons display a decreased sensitivity to NGF. *Development*.

[121] Reynolds B. A., Weiss S. (1992). Generation of neurons and astrocytes from isolated cells of the adult mammalian central nervous system. *Science*.

[122] Temple S. (1989). Division and differentiation of isolated CNS blast cells in microculture. *Nature*.

[123] Ramón-Cueto A., Avila J. (1998). Olfactory ensheathing glia: properties and function. *Brain Research Bulletin*.

[124] Park H.-W., Lim M.-J., Jung H., Lee S.-P., Paik K.-S., Chang M.-S. (2010). Human mesenchymal stem cell-derived Schwann cell-like cells exhibit neurotrophic effects, via distinct growth factor production, in a model of spinal cord injury. *Glia*.

[125] Ghosh M., Tuesta L. M., Puentes R. (2012). Extensive cell migration, axon regeneration, and improved function with polysialic acid-modified Schwann cells after spinal cord injury. *Glia*.

[126] Pierucci A., Duek E. A. R., De Oliveira A. L. R. (2009). Expression of basal lamina components by Schwann cells cultured on poly(lactic acid) (PLLA) and poly(caprolactone) (PCL) membranes. *Journal of Materials Science: Materials in Medicine*.

[127] Cyranoski D. (2005). Fetal-cell therapy: paper chase. *Nature*.

[128] Dobkin B. H., Curt A., Guest J. (2006). Cellular transplants in China: observational study from the largest human experiment in chronic spinal cord injury. *Neurorehabilitation and Neural Repair*.

[129] Curt A., Dietz V. (2005). Controversial treatments for spinal-cord injuries. *The Lancet*.

[130] Aznar J., Sánchez J. L. (2011). Embryonic stem cells: are useful in clinic treatments?. *Journal of Physiology and Biochemistry*.

[131] Sundberg M., Andersson P.-H., kesson E. A. (2011). Markers of pluripotency and differentiation in human neural precursor cells derived from embryonic stem cells and CNS tissue. *Cell Transplantation*.

